# Severe burn and disuse in the rat independently adversely impact body composition and adipokines

**DOI:** 10.1186/cc13048

**Published:** 2013-10-07

**Authors:** Charles E Wade, Lisa A Baer, Xiaowu Wu, David T Silliman, Thomas J Walters, Steven E Wolf

**Affiliations:** 1 US Army Institute of Surgical Research, Fort Sam Houston, TX 78234, USA; 2 Department of Surgery, University of Texas Health Science Center, San Antonio, TX 78229, USA; 3 Department of Surgery and Center for Translational Injury Research, University of Texas Health Science Center, 6431 Fannin St. MSB 5.204, Houston, TX 77030, USA

## Abstract

**Introduction:**

Severe trauma is accompanied by a period of hypermetabolism and disuse. In this study, a rat model was used to determine the effects of burn and disuse independently and in combination on body composition, food intake and adipokines.

**Methods:**

Male rats were assigned to four groups 1) sham ambulatory (SA), 2) sham hindlimb unloaded (SH), 3) 40% total body surface area full thickness scald burn ambulatory (BA) and 4) burn and hindlimb unloaded (BH). Animals designated to the SH and BH groups were placed in a tail traction system and their hindlimbs unloaded. Animals were followed for 14 days. Plasma, urine, fecal and tissue samples were analyzed.

**Results:**

SA had a progressive increase in body mass (BM), SH and BA no change and BH a reduction. Compared to SA, BM was reduced by 10% in both SH and BA and by 17% when combined in BH. Compared to SA, all groups had reductions in lean and fat body mass with BH being greater. The decrease in lean mass was associated with the rate of urinary corticosterone excretion. The loss in fat mass was associated with decreases in plasma leptin and adiponectin and an increase in ghrelin. Following the acute response to injury, BH had a greater food intake per 100 g BM. Food intake was associated with the levels of leptin, adiponectin and ghrelin.

**Conclusions:**

The effects of the combination of burn and disuse in this animal model were additive, therefore in assessing metabolic changes with severe trauma both injury and disuse should be considered. Furthermore, the observed changes in adipokines, corticosterone and ghrelin provide insights for interventions to attenuate the hypermetabolic state following injury, possibly reducing catabolism and muscle loss and subsequent adverse effects on recovery and function.

## Introduction

A major factor that impacts the treatment and recovery of patients with severe traumatic injuries is a pronounced increase in metabolism. Injury induces a systemic catabolic response characterized by increased energy expenditure and massive loss of body mass (BM). This substantial loss is due to reductions in both lean (LBM) and fat mass (FM) [1-7]. The loss of LBM subsequently compromises the patient’s ability to resume normal activities and has been attributed, in part, to prolonged inactivity associated with bed rest, as well as a response to injury [8]. Burns are suggested to induce the greatest increase in metabolic demand as a result of loss of the insulation properties of skin and the increased demands of wound healing. Hypermetabolism is evident immediately after injury, and peaks days after injury when oxygen and nutrient delivery to tissue increases, allowing for a further increase in metabolic activity [1]. The burn-induced imbalance of protein synthesis and breakdown is sustained from the time of hospital admission to discharge, when wounds are 95% or more healed. In some cases, catabolism persists for up to one year after discharge, resulting in substantial muscle wasting and atrophy during convalescence [2,3,6,9]. Patients with severe burns usually require long-term bed rest during hospitalization, and have limited mobility once leaving the hospital. Inactivity, coupled with increased metabolic demands, clearly contributes to a multitude of issues limiting and/or prolonging recovery from injury [10,11].

We, along with others, have recently reported alterations in plasma adipokines (leptin, adiponection and resistin) concentrations in burns and severe illness. Adipokines are derived from fat and have been associated with the inflammatory response to injury, insulin resistance, and the severity of illness. Adipokines have been extensively studied in metabolic diseases that have led to interest in their role in metabolic disorders observed with severe injury [12-16].

It has been observed that rats with full thickness burn ≥30% total body surface area (TBSA) have an acute hypermetabolic response similar to that reported in humans [7,17,18], persisting for over 14 days post injury [17-19]. The increase in metabolism was associated with a reduction in body protein and fat content when animals were held on a restricted diet [20,21]. When they were provided access to food *ad libitum*, their intake was increased [22,23]. This increased intake may alter the responses reported in body composition, and attenuate shifts in metabolic substrates. Furthermore, the question must be raised as to the possible role of disuse in these changes.

Hindlimb unloading (HLU) in rats has been commonly used to study the effect of inactivity on muscle metabolism and physiological changes of muscle due to disuse and the microgravity environment of space flight [24-27]. Importantly, HLU mimics the physiologic change of long term bed rest [24]. In addition, immunological alterations with inactivity may contribute to delayed wound healing, as well as potentiating the catabolic state [28,29]. Because of this, it is thought that HLU, in addition to burns, will more closely resemble the clinical condition in patients with severe burns.

To determine whether disuse is an additional or associate factor contributing to burn-induced hypermetabolism, and the loss of lean and fat body mass, we employed a rat model combining hindlimb unloading and severe burn.

## Material and methods

### Animals

All procedures were reviewed and approved by the US Army Institute for Surgical Research and University of Texas Health Science Center San Antonio Institutional Animal Care and Use Committees. Data from these experiments in regards to muscle function and bone health have been previously published [30,31].

Male Sprague-Dawley rats (250 to 275 g on arrival) were used for this study (Charles Rivers, Wilmington, MA, USA). Animals weighed approximately 300 g at the start of the experiment. All animals were given food and water *ad lib*. Light cycle 12:12 hr (0600, on:1800, off).

### Housing

Upon arrival, animals were housed in standard vivarium cages and then housed in specialized metabolic/unloading HLU cages (144 in^2^ usable floor area) one week before injury to allow for acclimation [32]. Animals were fed a certified diet (Harlan Teklad #2018), in powder-form, while housed in the HLU cages. Room temperature was maintained at 26 ± 2°C with 30 to 80% relative humidity to simulate, as closely as possible, the ambient temperature maintained in a standard burn unit.

### Experimental group assignments

Following 24 hours of data collection (Day 0), rats were randomly assigned to one of four experimental groups: Sham Ambulatory (SA; n = 10); Burn Ambulatory (BA; n = 9); Sham Hindlimb Unloading (SH; n = 10); Burn Hindlimb Unloading (BH; n = 10). A block design was used for this study where four animals were weight-matched to each other and then each animal was randomly assigned to one of the four treatment groups. This assignment was carried out prior to any experimental manipulations. Following the experimental treatments, animals were followed for 14 days.

### Animal husbandry measurements

Body masses of all animals were measured daily from the time of arrival until the end of the study. Animals assigned to SH and BH treatment groups were weighed using a hook attached to a ring-stand placed on the balance to avoid any type of weight-bearing on the hindlimbs during the weighing procedure [24]. Food and water intake were measured daily in all groups.

### Urine collection

Housing in the metabolic cages allowed for the collection of uncontaminated urine samples. Beginning one day before injury, 24-hour urine volumes were measured. Urine was aliquoted and samples were stored at -80°C for future analysis.

### Scald injury

Rats randomly assigned to either burn treatment group (that is, BA or BH) received a 40% total body surface area, full-thickness scald burn as described by Walker-Mason [33]. Rats were anesthetized with isoflurane (2 to 3% in 100% O_2_) throughout the procedure and administered buprenorphine (0.05 mg/kg s.c) prior to injury. Each rat was shaved and secured in a plexiglass mold exposing 20% of the total body surface area of the dorsal side. The dorsal surface was submerged in 100°C water for 10 seconds. The animal was removed from the mold, administered 20 cc of Lactated Ringer’s intraperitoneally, which was based on the modified Brooke Formula for resuscitation fluids in burn patients [34], placed back in the mold exposing the ventral (belly) surface and submerged in 100°C water for two seconds. Sham groups (SA and SH) were exposed to the anesthesia procedure, shaved and submerged in water at room temperature. All animals were administered additional analgesics (buprenorphine; 0.05 mg/kg s.c) 6 to 8 hours following the scald procedure and every 24 hours thereafter for 72 hours.

### Hindlimb unloading

Following the scald procedure described above, animals randomly assigned to the HLU group were placed in a tail traction system using an established HLU model [24]. Briefly, the tail was prepared for HLU by cleaning with alcohol wipes, tincture of benzoin was then applied, allowing it to become tacky to the touch. A half-inch strip of Skin Trac^©^ (Zimmer, San Jose, CA, USA) was secured on the tail, it was then wrapped in Stockinette, and three one-inch strips of filament fiber tape were applied (base, middle, top). Animals were allowed to completely recover from anesthesia, approximately 20 to 30 minutes, before being placed in HLU cages and their hindlimbs were unloaded at approximately 30° using a hook and pulley system. The pulley system allowed the animals to have 360° access within the cage environment without applying load to their hindlimbs. Animals were observed immediately after unloading for any apparent signs of distress and were monitored several times during the day throughout the study following the burn/HLU procedure.

### Tissue collection and body composition

At the conclusion of the study on Day 14, animals were deeply anesthetized with isoflurane (1 to 3% in 100% O_2_) and blood was collected via cardiac puncture. Animals were euthanized by exsanguination and predetermined tissues were harvested, weighed and minimal tissue samples obtained. Tissues measured were visceral fat (epididymal), liver and hindlimb muscles (*tibialis anterior, extensor digitorum longus, plantaris, soleus, medial gastrocnemius, lateral gastrocnemius*). Data as to individual hindlimb muscle masses have been previously presented [31]. Excess tissues were returned to the carcass prior to dual-energy X-ray absorptiometry (DEXA) measurements. DEXA scans were taken using Encore 2005 software (GE Lunar Prodigy, Madison, WI, USA) of all carcasses at the conclusion of the tissue collection to assess total body fat and lean body mass.

### Plasma assay

Plasma collected from whole blood samples were stored at -80°C for later analysis. Commercial plasma enzyme-linked-immunosorbent assays (ELISA; Linco Research, St. Charles, MO, USA; Neogen Corp, Lexington, KY, USA) were used to measure leptin (L), ghrelin (G), adiponectin (A), and resistin (R). Additional plasma samples were analyzed for blood urea nitrogen (BUN) and total protein (TP).

### Urine assay

Urine was collected daily, aliquoted and stored at -80°C for later analysis. Using commercial urinary ELISAs (Neogen Corp), daily urine samples were analyzed for corticosterone (CORT) and testosterone.

### Nitrogen balance

For nitrogen balance calculations, urinary urea nitrogen (UUN) was analyzed using a Dade Dimension Chemistry Analyzer® (Deerfield, IL, USA). Urinary nitrogen (N) was calculated for rats using duplicate determinations of UUN; urinary N (mg/mL) = (UUN (mg/mL) × 1.067) -0.302. Nitrogen intake was calculated as food intake x dietary protein content divided by 6.25. Fecal nitrogen was assumed to be a constant value of 60 mg/day. Nitrogen balance was calculated as the difference between N intake and N excretion (urinary + fecal) [35,36].

### Statistics

Two distinct patterns of metabolic change over the course of time have been identified in patients with burns and in animal models of injury [37,38]. Therefore, in the analysis of data over time, two periods were considered, the initial response to injury, the “ebb phase” lasting over three days and the compensatory “flow phase” after the initial response, days 3 to 14. Discontinuous data were compared using a Chi-square test. Continuous data were compared between groups using ANOVA and, where appropriate, adjusted for repeated measurements over time. A Tukey’s test was used for *post-hoc* analyses. Values in the text are reported as mean ± SEM and differences determined at *P* <0.05.

## Results

### Body mass

**Figure 1:**
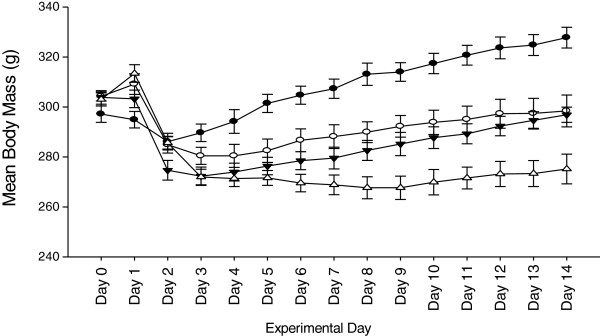
**Mean body mass over time.** No differences were observed between groups on Day 0. Significant differences were observed between treatment groups after Day 3 through Day 14; SA >BA = SH >BH, *P* <0.01 treatment effect. Sham-ambulatory (SA; closed circles); Burn-ambulatory (BA; open circles); Sham-hindlimb (SH; closed triangles); Burn-hindlimb (BH; open triangles).

There were no differences in initial body mass between the treatment groups; however, differences were apparent by Day 14 (Figure 1; Table 1). BA and SH showed similar reductions in body mass and BH had the most dramatic decrease over time, accentuated by an additive effect from the combination of burn and disuse. The percent changes in body mass over the 14 days were +10%, -2%, *-*3% and -10% for SA, BA, SH and BH, respectively.

**Table 1:** Physiological measurements

**Day 14**	**SA (N = 10)**	**BA (N = 9)**	**SH (N = 10)**	**BH (N = 10)**
Absolute body mass (g)	334.6 ±5.8	300.6 ± 2.4^a,d^	298.9 ± 3.4^a,d^	276.8 ± 11.7^a,c^
LBM (g)	238.7 ± 3.2	225.9 ± 4.7	218.9 ± 2.4	204.7 ± 5.1^a,b,c^
% LBM/BM	72.8 ± 0.6	75.7 ± 0.4	73.7 ± 0.5	74.3 ± 0.6
Fat mass (g)	51.5 ± 2.5	31.2 ± 1.7^a^	36.3 ± 1.6^a^	26.7 ± 1.5^a,c^
% Fat mass	15.7 ± 0.6	10.5 ± 0.6^a^	12.2 ± 0.5^a^	9.7 ± 0.5^a,c^
Visceral fat mass (g)	3.14 ± 0.13	2.13 ± 0.12^a,d^	2.01 ± 0.9^a,b,d^	1.47 ± 0.07^a,c^
Visceral fat mass/100 g BM (g)	0.96 ± 0.04	0.71 ± 0.03^a,d^	0.68 ± 0.03^a,d^	0.53 ± 0.02^a,c^
Liver mass (g)	10.12 ± 0.19	9.02 ±0.28^a,c^	8.14 ± 0.10^a,b^	8.31 ± 0.33^a^
Liver mass/100 g BM (g)	3.09 ± 0.04	3.03 ± 0.07	2.74 ± 0.04^a,b^	3.02 ± 0.11^b^
Total hindlimb muscle mass^π^ (g)	3.45 ± 0.07	3.00 ± 0.08^a,d^	2.71 ± 0.07^a,d^	2.33 ± 0.06^a,b,c^
Total hindlimb muscle mass/100 g BM (g)	0.85 ± 0.01	0.91 ± 0.02^a,b,d^	1.01 ± 0.02^a,c,d^	1.05 ± 0.02^a,b,c^

All values are those obtained on Day 14 of the experiment.

^a^Significantly different from SA (Sham Ambulatory), *P* <0.001; ^b^Significantly different from BA (Burn Ambulatory), *P* <0.001; ^c^Significantly different from SH (Sham Hindlimb), *P* <0.001;^d^Significantly different from BH (Burn Hindlimb), *P* <0.001. ^π^from a single leg. BM, body mass; LBM, lean body mass.

### Food consumption

**Figure 2:**
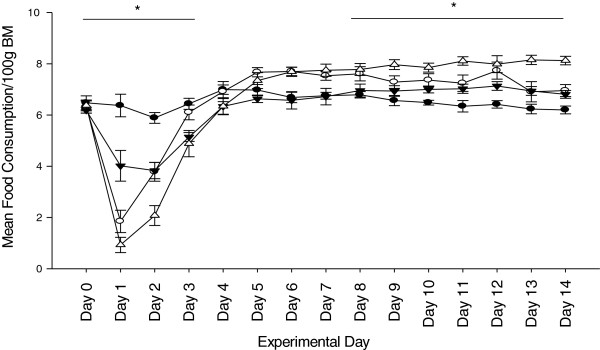
**Mean food consumption per 100 g of daily body mass over 14 days.** Independent analyzes were conducted during the “ebb phase” days 0 to 3, and the “flow phase” days 3 to 14. Significant differences were observed between SA and other treatment groups between Day 1 and Day 3 (SA >SH >BA = BH, *P* <0.01), and then from Day 9 until the end of the study (SA >SH = BA >BH, *P* <0.01). No differences were observed between BA and SH. Sham-ambulatory (SA; closed circles); Burn-ambulatory (BA; open circles); Sham-hindlimb (SH; closed triangles); Burn-hindlimb (BH; open triangles). * indicates significant differences between groups, *P* <0.05.

Over the course of the 14 days, there was no difference in total food consumption. However, there were significant differences in the food intake patterns, characteristic of the ebb and flow phases following injury. There was a significant reduction in absolute food consumption, even when corrected by body mass, on Day 1 in all treatment groups as compared to SA. After Day 1, food consumption slowly increased over time in all groups. When food consumption was corrected for body mass, a significant difference was observed between SA and the other treatment groups from Day 1 and Day 3. By Day 7 after injury, BH corrected food consumption was greater than the other treatment groups and from Day 9 on was significantly greater (*P* <0.001) than all treatment groups (Figure 2). Groups BA, SH and BH cumulative food intake corrected for body mass between days 3 to 14 was significantly changed from SA by 9.4%, 1.7% and 12.3%, respectively There were no differences observed between BA and SH.

### Lean body mass, fat mass, visceral fat mass, liver mass and total muscle mass

Using DEXA, there were significant differences in LBM measured between BH and all other treatment groups (Table 1). However, when LBM was corrected for body mass obtained on Day 14, there were no differences observed in percent LBM. There was a significant decrease in fat mass and percent fat mass in all treatment groups from SA (*P* <0.001), with the most dramatic reduction, 48%, being in BH group (Table 1). BH was significantly different from SH, but there were no differences observed between either of the HLU groups from BA, indicating that the burn injury may cause a physiological change that overwhelms the effect of the disuse.

There were significant reductions in visceral fat mass and visceral fat mass per 100 g BM in all groups as compared to SA. In addition, BA and SH were significantly different from BH, indicating that the loss in fat is independent in each manipulation, and additive when in combination (Table 1).

Liver mass was significantly reduced in all groups compared to SA. SH was significantly lower than BA, but BH was not different from BA or SH. When corrected for body mass, there were no differences between either of the burn groups, suggesting that in terms of liver mass, disuse alone has more of an overall effect than burn (Table 1).

Total hindlimb muscle mass, (the sum of mass of *tibialis anterior, extensor digitorum longus, plantaris, soleus, medial gastrocnemius, lateral gastrocnemius*), was significantly reduced in BH compared to all other groups. In addition, BA and SH were different from SA, but, were not different from each other, suggesting an additive effect on muscle mass.

### Urinary corticosterone and nitrogen balance over 14 days

**Figure 3:**
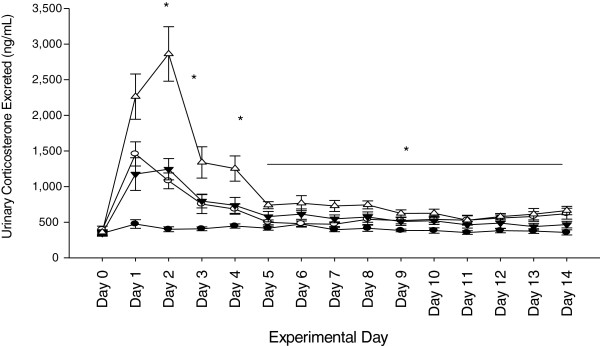
**Mean urinary corticosterone (CORT) over 14 days.** A significant increase in CORT was observed initially after treatment (BH >BA = SH >SA, *P* <0.001), but steadily declined by Day 14. Significant differences (*P* <0.001) were still apparent between BH and other treatment groups through Day 14. Sham-ambulatory (SA; closed circles); Burn-ambulatory (BA; open circles); Sham-hindlimb (SH; closed triangles); Burn-hindlimb (BH; open triangles). * indicates significant differences between groups, *P* <0.05.

Urinary CORT and testosterone concentrations were measured daily throughout the study. No differences in urinary CORT excretion rates were observed between any of the groups before the initiation of injury and HLU. A significant increase in CORT was observed between all groups from SA the day immediately following injury (*P* <0.001), with the most dramatic increase in BH. A steady decline was observed after Day 3; however, BH continued to be significantly higher than all other treatment groups. By Day 14, all groups remained significantly higher than SA (*P* <0.001); however, BA and SH showed similar responses by the end of the study (Figure 3). When average daily urinary CORT excretion was calculated, there were significant differences between BH and all treatment groups (*P* <0.001) (Table 2). There were no differences between BA and SH, indicating that in terms of total urinary CORT, the additive response had much more of an overall effect than each treatment alone. Average urinary testosterone was not different between groups (Table 2).

**Table 2:** Protein metabolism

**Day 14**	**SA (N = 10)**	**BA (N = 9)**	**SH (N = 10)**	**BH (N = 10)**
Mean urinary testosterone (ng/day)	2.95 ± 0.34	2.41 ± 0.30	2.57 ± 0.23	2.26 ± 0.26
Mean urinary corticosterone (ng/day)	377.7 ± 20.3	621.1 ± 35.1^a^	610.1 ± 62.7^a^	954.9 ± 55.9^a,b,c^
BUN (mg/dL)	25 ± 1	29 ± 1^a^	26 ± 1	35 ± 1^a,b,c^
Total protein (g/dL)	4.9 ± 0.1	4.8 ± 0.1	4.8 ± 0.1	4.9 ± 0.2

^a^Significantly different from SA (Sham Ambulatory), *P* <0.001; ^b^Significantly different from BA (Burn Ambulatory), *P* <0.001; ^c^Significantly different from SH (Sham Hindlimb), *P* <0.001;^d^Significantly different from BH (Burn Hindlimb), *P* <0.001.

**Figure 4:**
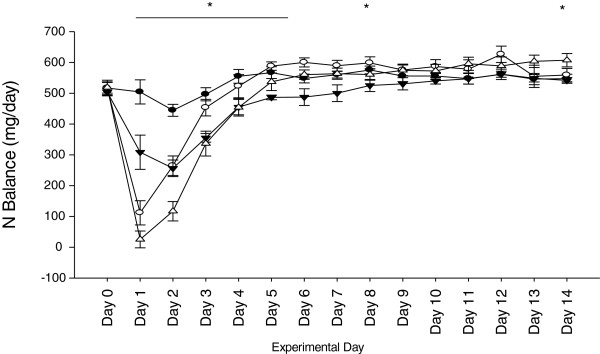
**Mean urinary nitrogen balance over 14 days.** The three treatment groups went into negative nitrogen balance after initial treatment (SA >SH = BA = BH, *P* <0.001), but a steady increase was observed over time. Significant differences were observed until Day 8 and then on the final day after treatment (SA >SH, *P* <0.001). Sham-ambulatory (SA; closed circles); Burn-ambulatory (BA; open circles); Sham-hindlimb (SH; closed triangles); Burn-hindlimb (BH; open triangles). * indicates significant differences between groups, *P* <0.05.

**Figure 5:**
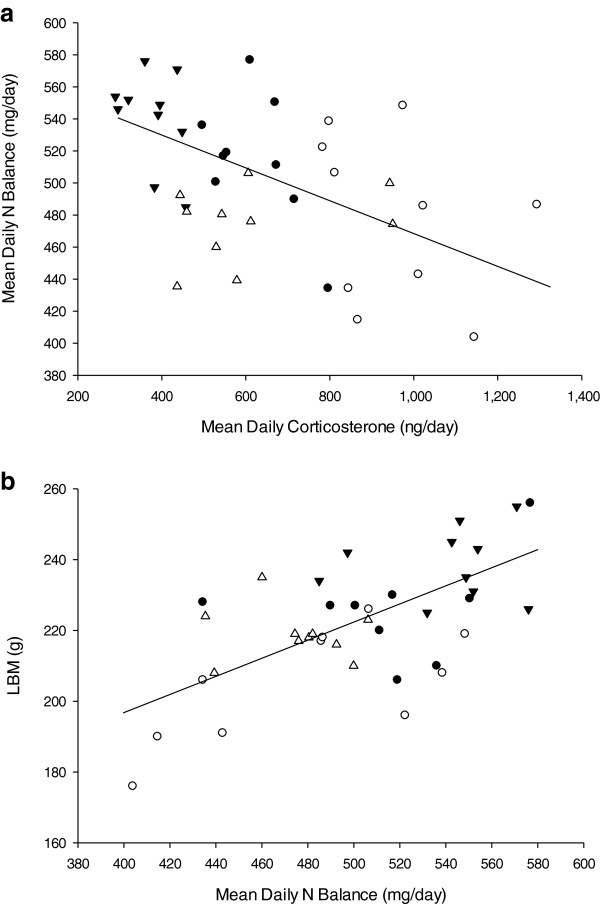
**Mean daily urinary nitrogen balance vs mean corticosterone and lean body mass. (a)** Mean daily urinary nitrogen balance from control vs mean daily urinary corticosterone. A correlation was observed between control vs daily urinary corticosterone (r = 0.47; *P* <0.003; n = 39). **(b)** Mean lean body mass (LBM) vs urinary nitrogen balance. A positive correlation was observed (r = 0.61; *P* <0.001; n = 39). Sham-ambulatory (SA; closed triangles); Burn-ambulatory (BA; closed circles); Sham-hindlimb (SH; open triangles); Burn-hindlimb (BH;, open circles).

Daily nitrogen balance was calculated for the duration of the study. No differences were observed between groups prior to injury. A significant reduction in nitrogen balance was observed within all groups the day following treatment, with both burn-treated groups showing the most dramatic response (*P* <0.001). Average daily nitrogen balance was different between groups (SA = 540.4 ± 9.2; BA = 515.0 ± 13.4; SH = 474.6 ± 7.5; BH = 478.4 ± 16.4 mg/day (*P* <0.001). There was a steady increase over the following seven days in all treatment groups. The increase in SH was not as great as the other treatment groups and was significantly lower than the other groups until the conclusion of the study (*P* <0.001) (Figure 4). The mean daily nitrogen balance was associated with the increases in average urinary CORT excretion over the same time (Figure 5a). A lower lean body mass on Day 14 was associated with a reduced daily nitrogen balance (Figure 5b). BUN concentrations were increased in all burn groups, while plasma total protein levels were not significantly altered (Table 2).

### Adipokines and ghrelin

**Figure 6:**
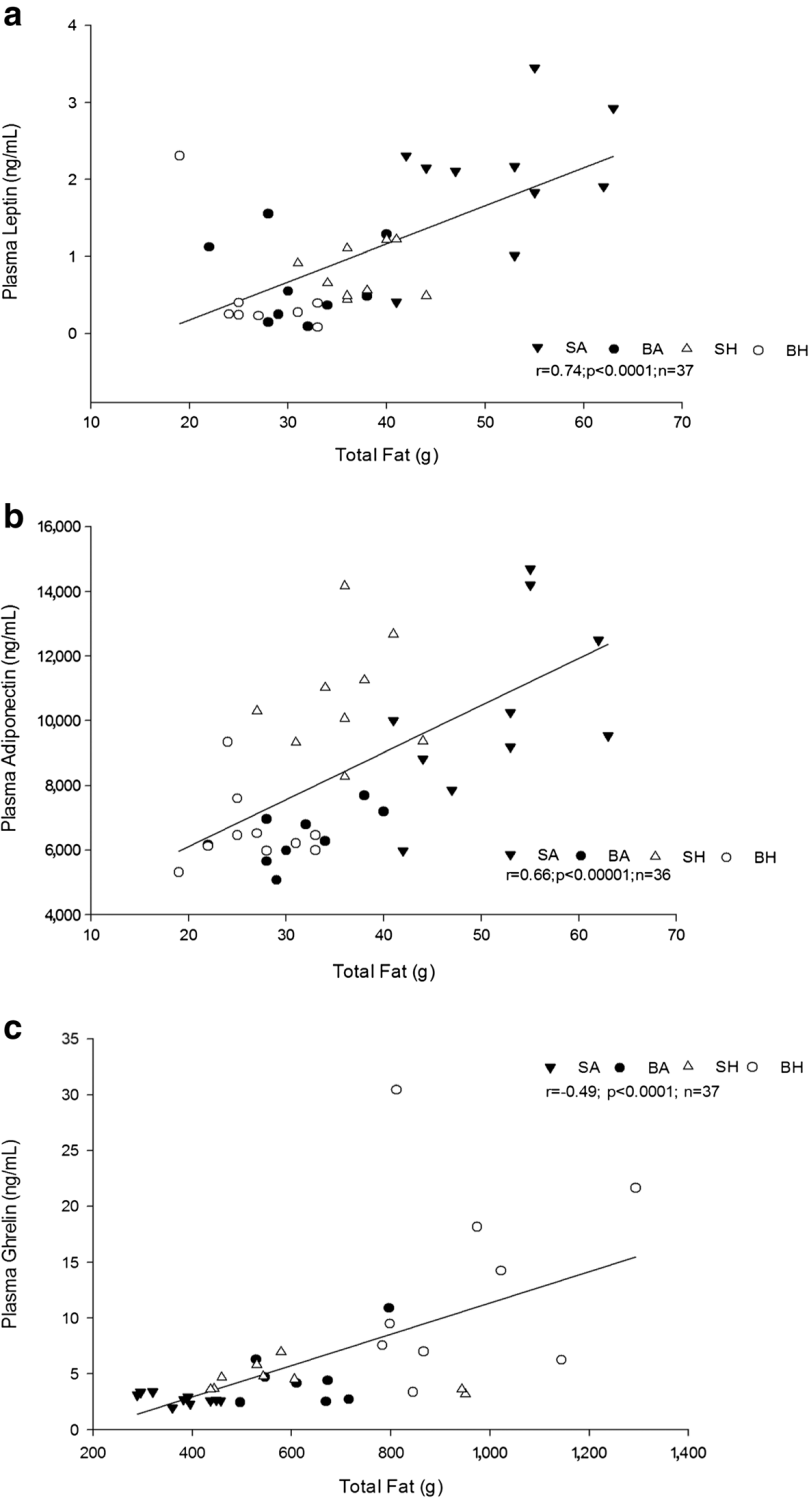
**Association of plasma leptin (a), adiponectin (b) and ghrelin (c) concentrations with body fat mass on Day 14. (a)** A positive correlation was observed between plasma leptin and total fat mass (r = 0.74; *P* <0.00001; n = 35). **(b)** A positive correlation was observed between plasma adiponectin and total fat mass (r = 0.61; *P* <0.0002; n = 36), **(c)** A negative correlation was observed between plasma ghrelin and total fat mass (r = -0.57; *P* <0.0002; n = 37). Sham-ambulatory (SA; closed triangles); Burn-ambulatory (BA; closed circles); Sham-hindlimb (SH; open triangles); Burn-hindlimb (BH; open circles).

To determine the effects of injury and disuse on fat metabolism, the primary adipokines, namely leptin, resistin and adiponectin, were analyzed, as well as the gut derived hormone ghrelin. Leptin, which plays a role in the regulation of energy intake and energy expenditure, had a profound effect due to injury and disuse after 14 days (Table 3). A significant decrease in plasma leptin was observed in all groups from SA (Table 3). A significant positive correlation was observed between plasma leptin and total body fat mass (r = 0.74, *P* <0.00001, n = 35) (Figure 6a).

**Table 3:** Fat metabolism

**Day 14**	**SA (N = 10)**	**BA (N = 9)**	**SH (N = 10)**	**BH (N = 10)**
Plasma leptin (ng/mL)	2.21 ± 0.2	0.57 ± 0.18	0.82 ± 0.11^a^	0.27 ± 0.04^a,b,c^
Plasma ghrelin (ng/mL)	2.8 ± 0.2	4.8 ± 0.9	4.5 ± 0.4	14.7 ± 2.7^a,b,c^
Plasma adiponectin (ng/mL)	10305 ± 916	6425 ± 289^a,c^	10714 ± 606^a,d^	6671 ± 390^a,c^
Plasma resistin (ng/mL)	10.8 ± 1.3	8.5 ± 0.9	14.5 ± 1.3^b,c^	9.7 ± 0.8

^a^Significantly different from SA (Sham Ambulatory), *P* <0.001; ^b^Significantly different from BA (Burn Ambulatory), *P* <0.001; ^c^Significantly different from SH (Sham Hindlimb), *P* <0.001;^d^Significantly different from BH (Burn Hindlimb), *P* <0.001.

Adiponectin, a protein hormone, modulates glucose regulation and fatty acid catabolism. Significant differences were observed between the burn treatment groups from SA (Table 3). The disuse group was not different from SA, indicating that the burn injury component has significant effects on adiponectin, rather than the disuse component. Like leptin, there was a significant positive correlation between adiponectin and total body fat mass (r = 0.61, *P* <0.0002, n = 35) (Figure 6b).

Plasma resistin was increased only in the SH group compared to the burn groups (Table 3).

Ghrelin, considered the counterpart of leptin, stimulates appetite rather than causing an increase in metabolism. A significant increase in plasma ghrelin was observed in the BH group compared to the other groups (Table 3). No significant differences were observed between SA, BA or SH. A negative relationship was observed between plasma ghrelin and total body fat mass (r = -0.57, *P* <0.0002, n = 35) (Figure 6c).

Food intake adjusted for body mass on Day 14 was associated with changes in leptin, adiponectin and ghrelin. Lower levels of leptin (r = -0.47; *P* <0.004; n = 37) and adiponectin (r = -0.49; *P* <0.002; n = 37) were moderately associated with increased food intake. In contrast, food intake was related to increased concentrations of ghrelin (r = 0.46; *P* <0.001; n = 36).

## Discussion

Hypermetabolism and catabolism are an on-going concern in the care of patients with severe injuries that necessitate bed rest [1,4,7,37,39-42]. Several factors associated with injury are a result of the hypermetabolic response. In the present study of burn and disuse, there was a reduction in body mass with an increase in food consumption three days after injury, obvious indices of a hypermetabolic state associated with the flow phase. We employed an animal model incorporating severe burn and disuse independently and in combination. In the majority of metabolic parameters measured, the effects were additive, suggesting independent contributions of burn and disuse to the shifts observed in patients (Table 4). Burn and disuse independently decreased body mass on Day 14 by 10.7% and 10.2%, respectively, but resulted in a 17.3% decrease when in combination (Table 4). Similar observations were made for lean and fat mass; however, the proportional decrease in fat mass was greater, representing a larger contribution to the reduction in body mass. This disproportionally greater decrease in fat mass is similar to that observed in severely burned patients. Additionally of interest, was the additive effect noted in corticosterone. Previous studies have shown that the response to additive stresses does not result in higher levels, but persistently elevated levels, which is in contrast to the additive effect observed in the present study. Historically, studies of burn in rat models do not usually account for the effects of disuse. Shangraw and Turinsky conducted studies of combine burn and disuse in rats by casting the hind legs, but noted no effect of disuse [43,44]. Wolfe and colleagues have conducted extensive metabolic studies in humans during bed rest and inferred effects of the patient with traumatic injuries [42]. Our study is the first to demonstrate the effects in combination and shows the lack of interaction between the two treatments and that in many cases their effects are additive. Thus, as proposed by Wolfe *et al*., injury and disuse need to be considered independently in the evaluation of interventions for the treatment of the critically injured patient [42,45].

**Table 4:** Percent change from control (SA)

	**BA**	**SH**	**BH**
Body mass	-10%	-10%	-17%
Lean body mass	-5%	-8%	-14%
Fat mass	-39%	-30%	-48%
Food intake	12%	4%	17%
Nitrogen balance	-5%	-12%	-11%
Urinary corticosterone	64%	61%	153%

BA, Burn Ambulatory; BH, Burn Hindlimb; SH, Sham Hindlimb.

The loss of lean body mass was associated with a net negative nitrogen balance, increased BUN and decreases in hindlimb muscle mass, which are greatest in the combination of burn and disuse. A catabolic state was persistent in animals with BH with both injury and disuse contributing. Shangraw and Turinsky evaluated protein turnover in the soleus muscles of burned (6 to 7% TBSA) rats whose hindlimbs were casted [43,44]. In contrast to the present study, they found disuse to be only a minor contributor. However, they reported no change to disuse alone, while we and others have demonstrated extensive reductions in our HLU model, as well as bed rest in humans [24,46,47]. The overall catabolism, associated with a reduction in lean mass that we observed is similar to that in a patient in negative nitrogen balance, with decreased immunologic function and all of the wound-healing problems associated with protein loss and malnutrition. In the present study, the decrease in lean mass was related to corticosterone in the absence of a change in testosterone. Ferrando and colleagues found that bed rest sensitized skeletal muscles to the catabolic effects of cortisol [40,48]. However, in the present study of injury and/or disuse, the effect of increased corticosterone levels was linear with an additive effect of the two manipulations noted indicative of no change in sensitivity.

Decreases in body and visceral fat masses were associated with significant decreases in plasma leptin and adiponectin concentrations. Both leptin and adiponectin have recently been shown to be reduced in critically ill patients and associated with stress related parameters [15,49,50]. We have recently seen similar changes in adipokines in patients with severe burns [16]. These adipokines have been suggested to play important roles in tissue inflammation; however, a role in critically ill patients has yet to be elucidated [51-53]. In the present study, reduced adipokine levels were observed in the presence of injury rather than disuse, suggesting a greater influence of the injury component. Administration of leptin in burned rats has been shown to increase angiogenesis in injured tissues and decrease multiple organ damage [54,55]. Resistin was not altered in the present studies, but has been reported to be decreased in children with burns [56]. In contrast, an increase in resistin is noted in patients in the intensive care unit who are septic [13]. Recently, days after burn injury resistin levels have been reported to be increased and associated with plasma cortisol concentration and with the level of insulin resistance [16,57].

A unique observation in the present study is the negative association of adiponectin and leptin with body fat mass. In normal subjects, these adipokines are inversely associated with body fat, as body fat decreases, adiponectin levels increase and leptin concentrations are reduced [51,58-60]. The decrease in both adipokines associated with the reduction in body fat mass in animals with burns and disuse raises questions as to secondary mediation possibly via cytokines or catecholamines. Lower levels of adiponectin are also associated with insulin insensitivity and increased hepatic fat content which are often reported in patients with severe burns [51].

Of note is the increase in food intake in the presence of an elevation in ghrelin in animals with both burn and disuse. The increase in this orexigenic hormone again is reflective of the hypermetabolic state post injury [60]. Recent work has suggested ghrelin administration during the acute or “ebb” phase in burned rats improves outcome by increasing growth hormone, inhibiting protein breakdown and attenuating inflammatory processes [61-63]. In the present study, measurements were taken on Day 14 after injury during the late or “flow phase”. The alteration of ghrelin and adipokines with burn and disuse offers possibilities for interventions to attenuate the persistent hypermetabolism of patients with severe traumatic injuries.

The present animal model incorporates burn injury and disuse; however, in patients there are additional manipulations affecting outcomes. These include early excision and grafting. The impact of these procedures and other clinical manipulations, such as wound care, could exasperate and prolong the findings observed in the present study during the ebb phase, necessitating a greater compensatory response during the flow phase. Another limitation in the inference of these data to the human condition is metabolic differences. For example, in rats the rate of muscle protein synthesis is approximately 2.5-fold greater than that of humans. That said, the dynamic changes in response to burn appear to be similar between the present animal model and the patient with severe burns [64].

## Conclusions

Clinically, our animal model of injury and disuse allows examination of the dramatic metabolic changes that occur due to trauma. Independently, burn injury and disuse had similar body mass reductions from control; however, when combined, additive effects were apparent. We have previously demonstrated, using this model, a similar response of an additive effect of burn and disuse on muscle function and bone health [30,31]. Thus, in assessing metabolic changes with severe trauma both injury and disuse should be considered. Furthermore, the observed changes in corticosterone, adipokines and ghrelin provide insights for interventions to attenuate the hypermetabolic state following injury, possibly reducing catabolism and muscle loss and the subsequent adverse effects on recovery and function.

## Key messages

• We evaluated independent and combined effects of burn and disuse on metabolism in a rat model.

• Lean and fat body mass was reduced in all groups compared to sham ambulatory animals.

• Decrease in lean body mass was associated with the rate of urinary corticosterone excretion.

• Loss of fat body mass was associated with decreased plasma leptin and adiponectin levels, and increased ghrelin levels.

• Burn and disuse produced additive effects on metabolic changes in this animal model.

## Abbreviations

A: Adiponectin; BA: Burn ambulatory; BH: Burn hindlimb unloading; BM: Body mass; BUN: Blood urea nitrogen; CORT: Corticosterone; DEXA: Dual energy x-ray absorptiometry; ELISA: Enzyme linked immunosorbent assay; FM: Fat mass; G: Ghrelin; HLU: Hindlimb unloading; L: Leptin; LBM: Lean body mass; N: Nitrogen; R: Resistin; SA: Sham ambulatory; SH: Sham hindlimb unloading; TBSA: Total body surface area; TP: Total protein; UUN: Urinary urea nitrogen.

## Competing interests

The authors have no competing interests to declare. The opinions or assertions contained herein are the private views of the authors and are not to be construed as official or reflecting the views of the US Department of Defense or the US Government. Some of the authors are employees of the US Government. This work was prepared as part of their official duties and, as such, there is no copyright to be transferred.

## Authors’ contributions

CEW, LAB, XW, TJW and SEW designed the study. CEW, LAB, XW and DTS performed animal work and adipokine measurements. CEW, LAB, XW and SEW analyzed the data. CEW, LAB, XW, DTS, TJW and SEW drafted the manuscript. All authors critically revised the manuscript and approved the manuscript for submission.
